# The relationship between alcohol consumption and outcomes after gastrointestinal surgery: a systematic review and meta-analysis

**DOI:** 10.1093/alcalc/agaf002

**Published:** 2025-02-05

**Authors:** Rebecca Angus, Tjun Wei Leow, David Humes, Alfred Adiamah

**Affiliations:** Gastrointestinal Surgery, Nottingham Digestive Diseases Centre and National Institute for Health Research Nottingham Biomedical Research Centre, Nottingham University Hospitals NHS Trust and University of Nottingham, Queen's Medical Centre, Nottingham NG7 2UH, United Kingdom; Gastrointestinal Surgery, Nottingham Digestive Diseases Centre and National Institute for Health Research Nottingham Biomedical Research Centre, Nottingham University Hospitals NHS Trust and University of Nottingham, Queen's Medical Centre, Nottingham NG7 2UH, United Kingdom; Gastrointestinal Surgery, Nottingham Digestive Diseases Centre and National Institute for Health Research Nottingham Biomedical Research Centre, Nottingham University Hospitals NHS Trust and University of Nottingham, Queen's Medical Centre, Nottingham NG7 2UH, United Kingdom; Gastrointestinal Surgery, Nottingham Digestive Diseases Centre and National Institute for Health Research Nottingham Biomedical Research Centre, Nottingham University Hospitals NHS Trust and University of Nottingham, Queen's Medical Centre, Nottingham NG7 2UH, United Kingdom

**Keywords:** gastrointestinal surgery, postoperative outcomes, alcohol consumption

## Abstract

The study aimed to summarise the evidence of the association between preoperative alcohol consumption and postoperative complications in gastrointestinal surgeries. Comprehensive searches of MEDLINE, EMBASE, and Cochrane databases were undertaken to identify original studies investigating the association between preoperative alcohol consumption and postoperative complications occurring within 30 days of surgery. The primary outcome was 30-day mortality risk and secondary outcomes included postoperative complications such as surgical site infections and risk of anastomotic leak. The pooled odds ratios (ORs) and 95% confidence intervals (CIs) were estimated using a random effects model. In total, 3601 reports were identified and reviewed for eligibility, then data was extracted from 26 studies that met inclusion criteria. 13 studies were included in the meta-analysis. The total number of patients in the meta-analysis was 686 181 including 20 163 with a high alcohol intake. Clearly defined high preoperative alcohol consumption was associated with an increased risk of postoperative complications including 30-day mortality (OR = 1.56; 95% CI: 1.07–2.28). The risk of anastomotic leak was significantly increased in those undergoing colorectal surgery with a high alcohol intake, OR 2.17 (95% CI: 1.74–2.72). An increase in risk was also found for surgical site infections in those undergoing gastrointestinal surgery with high alcohol intake. (OR = 1.32; 95% CI: 1.15–1.53). Preoperative alcohol consumption was associated with an increased risk of 30-day mortality, anastomotic leak and surgical site infections. Preoperative modulation of alcohol intake may influence post-operative complications after gastrointestinal surgery.

## Introduction

Alcohol is a major contributor to global morbidity and is responsible for > 3 million deaths a year worldwide (Harmful use of alcohol [Internet] 2025). It is recognised by the World Health Organization as one of the four major causes of non-communicable diseases (Noncommunicable diseases [Internet] 2025). Against the backdrop of an already rising incidence of alcohol-use disorders, the COVID-19 pandemic further augmented alcohol misuse, with findings that suggested those with problematic drinking habits were drinking significantly more (Grossman et al. 2020). One of the many consequences of alcohol misuse is an increased risk of gastrointestinal diseases, which can ultimately lead to the need for gastrointestinal surgery (Bode and Bode 1997). Preoperative alcohol consumption is an identifiable and targetable modifiable risk factor, therefore understanding the impact of alcohol on postoperative outcomes after gastrointestinal surgery makes this review particularly relevant.

An earlier systematic review published in 2013 suggested that preoperative alcohol use increased postoperative complications across all surgery types (Eliasen et al. 2013). However, gastrointestinal procedures were under-represented in the review. Since then, there has been no review that specifically evaluates outcomes after gastrointestinal surgery in the context of problem drinking and clear guidelines or protocols on alcohol cessation prior to surgery are lacking. Alcohol is responsible for 5.1% of all disease and injury burden worldwide (Harmful use of alcohol [Internet] 2025). Alcohol increases the endocrine stress response, reduces the immune system efficiency, and decreases the effectiveness of certain drugs (Spies et al. 2004; Tsuchiya 2016). These properties affect how the body responds to surgery and its capacity to recover from it.

The aim of this systematic review was to summarise the existing literature on pre-operative alcohol consumption and postoperative outcomes following gastrointestinal surgery.

## Methods

The systematic review was conducted according to the Preferred Reporting Items for Systematic Reviews and Meta-Analysis (PRISMA) statement.

### Literature search

Systematic electronic searches were performed using the OVID platform to search the Embase and Medline electronic databases, from 1996 and 1980 respectively, to October 2023. The Cochrane Library was also searched, to identify all studies reporting on alcohol consumption and postoperative gastrointestinal complications. The detailed search strategy is presented in the supplementary material Appendix 1. The search terms included [‘alcohol’ ‘harmful drinking’ ‘alcohol misuse’] AND [‘gastrointestinal surgery’ ‘abdominal surgery’] AND [‘postoperative outcomes’ ‘morbidity’ ‘mortality’]. Handsearches of reference lists of relevant studies were undertaken to ensure comprehensive inclusion.

### Inclusion criteria

The titles and abstracts of studies identified from the searches were screened for suitability independently by two authors (RA and TWL) and any discordant articles were adjudicated by a third author (DH). For a study to meet the eligibility criteria, it had to report on human adult subjects who were undergoing gastrointestinal surgery and also report on their alcohol consumption. Our inclusion criteria for data extraction for the systematic reveiew was any consumption of alcohol.

### Exclusion criteria

Studies including children or adolescents were excluded. Animal studies, case reports, case series (defined as the number of study participants <10), letters, comments and editorials were excluded. No language limitation was applied.

### Data extraction

Two reviewers independently extracted data (RA and TWL) into predefined templates and this was adjudicated by a third reviewer (DJH) to resolve any discrepancies. The following data was extracted: characteristics of the study (author, country of origin and year of publication), study design, the quantity of overall population and alcohol-exposed population, measurement of alcohol consumption, type of surgery, description of postoperative complications and the rate or odds ratio of the association between alcohol consumption and postoperative complications.

### Risk of bias

The risk of bias assessment was analysed independently by two review authors (RA and TWL). For non-randomized studies, the Newcastle-Ottawa Scale quality assessment was used and for randomized controlled trials (RCT) the Cochrane Collaboration tool was used. All studies are reported as low, moderate, or high risk of bias and are presented using the Cochrane risk of bias traffic light plot.

### Statistical analysis

The primary outcome was the risk of 30-day mortality, which was defined as all deaths recorded within the first 30-days after the index surgical procedure. Secondary outcomes included surgical site infections (SSIs), which were defined as any reported infections at the operative wound site – that occurred within 30-days of surgery, irrespective of Clavien-Dindo grade. Finally, anastomotic leak was defined directly from the individual studies – as all grades of failure of anastomotic joins were reported – irrespective of the treatment of the anastomotic leak.

The exposure variable in this study was defined as a high alcohol intake. In the systematic review all studies which a description of ‘high alcohol intake’ were included. However, for the purpose of the meta-analysis, our inclusion criteria was more clearly defined as greater than 14 units a week, as per the European low risk alcohol intake measure which is also commensurate with the recommended threshold of a weekly alcohol consumption of < 14 units in the United Kingdom. (National low-risk drinking recommendations (or drinking guidelines) and standard units | Knowledge for policy [Internet] 2025; Drink less - Better Health - NHS [Internet] 2025).

For pooling of data, we used either the effect estimates as provided by the individual studies where feasible or, as an alternative, calculated the total number of events over total number in the ‘no alcohol’ and ‘high alcohol’ intake groups to generate the odds ratio of the event. The log odds ratio and log of the standard error (SE) from all relevant studies were then pooled to estimate the overall odds ratio of 30- day mortality, anastomotic leaks, and surgical site infections between patients with high alcohol intake and no alcohol intake using the inverse variance method assuming a random effects model. The inverse variance approach assigns a weight that is equal to the inverse of the variance of the effect estimate. Therefore, larger studies with smaller standard errors, are given more weight than smaller studies with larger standard errors. This choice of weighting minimizes the imprecision (uncertainty) of the pooled effect estimate. (9.4.3.0 Introductory text [Internet] 2025).

The amount of statistical heterogeneity of the included studies was evaluated using the *I*^2^ statistic. An *I*^2^ value between 0% to 40% may not be important, 30% to 60% may represent moderate heterogeneity, 50% to 90% may represent substantial heterogeneity and 75% to 100% indicates considerable heterogeneity. All data analysis were performed using Stata® version 16 (StataCorp, College Station, TX).

### Publication bias

Publication bias was assessed as per the methods described by Egger et al using the visual inspection for asymmetry of the funnel plot.(Identifying publication bias in meta-analyses of continuous outcomes | Cochrane Training [Internet] 2025).

### Protocol registration

The protocol was registered with the PROSPERO database (www.crd.york.ac.uk/prospero)—registration no. CRD42022366879 on the 10 October 2022.

## Results

**Figure 1:**
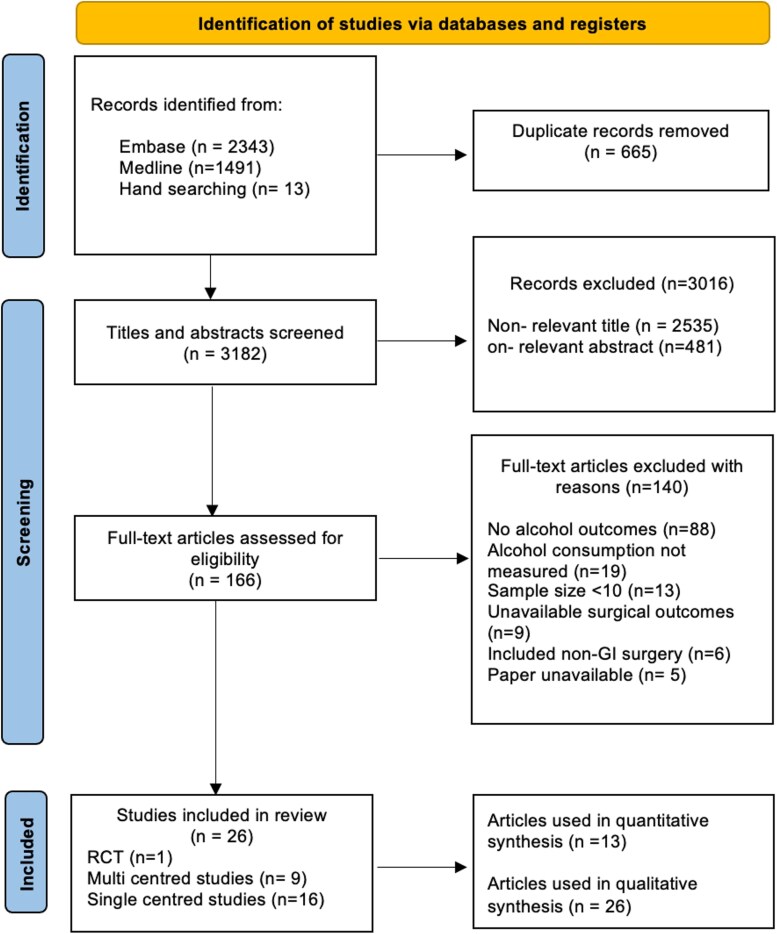
PRISMA flow diagram that maps out the identification of papers.

Of the 3601 studies identified, 166 full-text articles were evaluated of which 26 studies met the inclusion criteria. Of these, 13 studies provided relevant data used within the quantitative analysis as shown in Fig. 1.

Six of these studies originated from America, five from Germany, four each from China and Denmark, two from Romania, and a single study each from Italy, Japan, Finland, and Turkey. The total number of patients in the included studies in the meta-analysis was 686 181 including 20 163 with a high alcohol intake. The operative procedures undertaken in these studies were performed on the oesophagus, stomach, pancreas (grouped as upper gastrointestinal) in a total of 12 studies, with another 14 studies on colorectal resectional surgery.

### Alcohol consumption

The individual studies quantified alcohol intake in different ways, including: units/day, units/week, total number of drinks/day, and grams of alcohol per day or per week. They also reported different thresholds of high intake.

Five studies employed a threshold of >60 g (7.5 units) of ethanol a day (Tønnesen et al. 1987; Tønnesen et al. 1999; Sander et al. 2002; Spies et al. 2004; Nickelsen et al. 2005) to define high alcohol intake. Four studies simply reported ‘alcohol use’ (Mäkelä et al. 2003; Kocer et al. 2007; Jannasch et al. 2015; Almussallam et al. 2016) with no distinct cut off, two studies used a diagnosis of an alcohol use disorder (Kulshrestha et al. 2021; Rolfzen et al. 2022), and two used >2 drinks a day (Molena et al. 2014; Turrentine et al. 2015) as their definition. The rest of the studies had distinctive thresholds of 17.5 units a week (Iancu et al., 2008), excessive intake (Stumpf et al. 2009), ‘ever vs never’ had a drink (Zhou et al. 2018), >2 units a day (Xing et al. 2021), alcohol abuse (Pop et al. 2018), >4 units a day (Bailey et al. 2003), >1 drink a day (Patti et al. 2011), >35 units a week (Bertelsen et al. 2010), >40 units years (Hidaka et al. 2007), drinking >1 time a week (Huang et al. 2021), >48 units a week (Sørensen et al. 1999), >1 drink a week (Sun et al. 2016), and a diagnosis of chronic alcoholism (Spies et al. 1996).

The majority of thresholds are comparable to the EU low alcohol consumption threshold of less than 14 units a week however, ‘alcohol use’, ‘ever vs never’ and ‘drinking more than once a week’ were ambiguous and did not state if these fitted above or below these drinking guidelines. This variability and heterogeneity in the clinical definitions of high alcohol intake limits aggregation of outcomes when pooling results.

### Description of complications

Across the 26 studies, five reported our primary outcome of 30-day mortality in patients undergoing both colorectal and upper gastrointestinal surgery. Six reported the risk of anastomotic leak in colorectal surgery and five reported on surgical site infection. Table 1 describes the studies and the postoperative complications reported. A meta-analysis was conducted to examine the outcomes of 30-day mortality, anastomotic leak, and surgical site infection. These specific outcomes were selected because they were the only ones reported in an adequate number of papers, which allowed for a meaningful analysis.

**Table 1:** Characteristics of the included studies.

**Author (year)**	**Country**	**Database**	**Study time frame**	**Overall population**	**Alcohol population**	**Exposure**	**Surgery**	**30-day mortality**	**Other complications**
**Randomised control trials**									
(Tønnesen et al. 1999)	Denmark	–	1995–1998	42	19	>7.5 units/day	Colorectal	Yes	Wound infection, wound haematoma, pneumonia, thrombophlebitis, UTI, anastomotic leakage, sepsis, psychosis
**Population-based cohort /multi-centre studies**									
(Bertelsen et al. 2010)	Denmark	DCCR	2001–2004	1495	35	>35 units/week	Colorectal	No	Anastomotic leak
(Rolfzen et al. 2022)	America	PHD	2016–2019	603 730	17 308	Alcohol use disorder	Colorectal	Yes	Length of stay
(Patti et al. 2011)	Italy		2007–2009	100	17	>1 drink a day	Colorectal	No	Delirium
(Nickelsen et al. 2005)	Denmark	DCCG	2001–2002	5181	64	>7.5 units/day	Colorectal	Yes	Deep wound infection, thrombosis, impaired wound healing, and anastomotic leak
(Jannasch et al. 2015)	Germany	QACC	2000–2010	17 867	347	Use or no use	Colorectal	No	Anastomotic leak
(Bailey et al. 2003)	America	VA-NSQIP	1999–2001	1777	341	>4 units/day	Upper	Yes	–
(Molena et al. 2014)	America	NSQIP	2005–20,012	2945	97	>2 drinks a day	Upper	Yes	Respiratory complication
(Kulshrestha et al. 2021)	America	NSQIP	2012–2015	59 490	2060	NIS classification of AUD	Upper and HPB	Yes	Length of stay, myocardial infarction, hypotension, pneumonia, acute respiratory failure, infection, nausea and vomiting, acute renal failure, UTI, SSI, DVT, blood transfusions, postoperative fistula, gastrointestinal complications
(Turrentine et al. 2015)	America	NSQIP	2003–2006	7030	1019	>2 drinks a day	All GI	No	Anastomotic leak
**Population single-centred studies**									
(Sørensen et al. 1999)	Denmark	–	1993–1996	333	8	>48 units/week	Colorectal	No	Anastomotic leak
(Pop et al. 2018)	Romania	–	2013–2015	605	100	Alcohol abuse	Colorectal	No	1 year mortality, anastomotic fistula, intra-abdominal abscess, postoperative haemorrhage, postoperative ileus, cardiac complications, pulmonary thromboembolism, and acute renal failure
(Zhou et al. 2018)	China	–	2010–2014	956	39	Ever vs never	Colorectal	No	Anastomotic leak
(Almussallam et al. 2016)	America	–	2007–2011	4879	2981	Any use of alcohol	Colorectal	No	30-day readmission
(Iancu et al., 2008)	Romania	–	2002–2006	993	103	17.5 units/week	Colorectal	No	Anastomotic leak
(Tønnesen et al. 1987)	Denmark	–	1976–1984	279	32	>7.5 units/day	Colorectal	Yes	Wound infection, pneumonia, wound rupture, bleeding, anastomotic leakage, pulmonary embolism, delirium
(Mäkelä et al. 2003)	Finland	–	1992–2001	88	20	Use of alcohol	Colorectal	No	Anastomotic leak
(Stumpf et al. 2009)	Germany	–		400	76	Excessive intake	Colorectal	No	Anastomotic leak
(Xing et al. 2021)	China	–	2009–2019	398	80	>2 units/day	Upper	No	Anastomotic leak
(Hidaka et al. 2007)	Japan	–	1978–2005	185	159	>40-unit years	Upper	No	5-year overall survival
(Huang et al. 2021)	China	–	2014–2017	615	225	Drinking >1 time /week	Upper	No	5-year overall survival
(Sander et al. 2002)	Germany	–	2001–2002	45	25	>7.5 units/day	Upper	Yes	Pneumonia, sepsis, and length of stay
(Spies et al. 2004)	Germany	–	2004	54	31	>7.5 units/day	Upper	No	Length of stay, infection, pneumonia, urinary tract infection, wound infection
(Kocer et al. 2007)	Turkey	–	2001–2004	269	33	Present or absence	Upper	Yes	–

DCCR = Danish Colorectal Cancer Register, PHD = Premier Healthcare Database, DCCG = Danish colorectal cancer group, QACC = International Quality Assessment Study in Colorectal Cancer, VA-NSQIP = Veterans Affairs National Surgical Quality Improvement Program, NSQIP = National Surgical Quality Improvement Program, NIS = network and information systems, AUD = alcohol use disorder, UTI = Urinary tract infections, SSI = surgical site infection, DVT = Deep vein thrombosis

### Meta-analysis

#### Primary outcome: 30-day mortality

**Figure 2:**
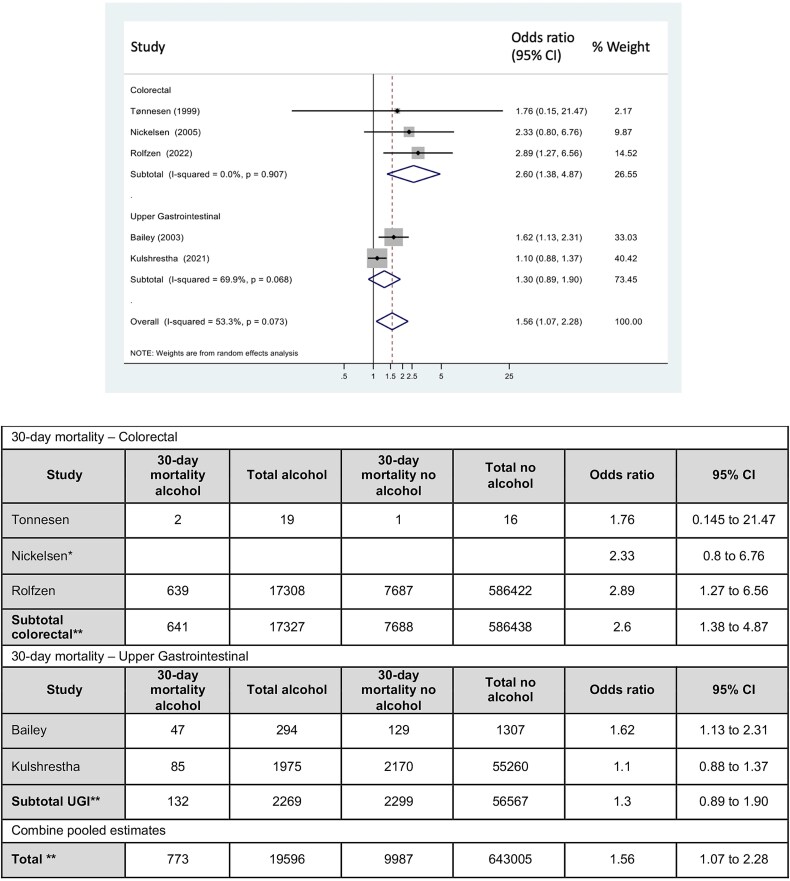
Forest plot for the association between preoperative alcohol consumption and 30-day mortality rate. There are two subgroups Upper Gastrointestinal and colorectal surgeries. Heterogeneity was assessed using the I^2^ statistics with the associated P value (P). ^*^ denotes studies which report only event rate (Nickelsen et al). ^**^ Subtotal in Upper Gastrointestinal and colorectal subgroups; and ^**^ total - only include studies that provide actual events; apart from odds ratio which is a combined pooled estimate.

The primary outcome of 30-day mortality was reported by five studies (Tønnesen et al. 1999; Bailey et al. 2003; Nickelsen et al. 2005; Kulshrestha et al. 2021; Rolfzen et al. 2022) on colorectal and upper gastrointestinal surgery. Clearly defined high preoperative alcohol consumption was associated with an overall increased risk of 30-day mortality (OR = 1.56; 95% CI: 1.07–2.28, I^2^ = 53.3%), Fig. 2. However, subgroup analysis showed a significant result only for those undergoing colorectal resection with an OR 2.60 (95% CI: 1.38–4.87, *I*^2^ = 0%).

### Secondary outcomes

#### Anastomotic leak

**Figure 3:**
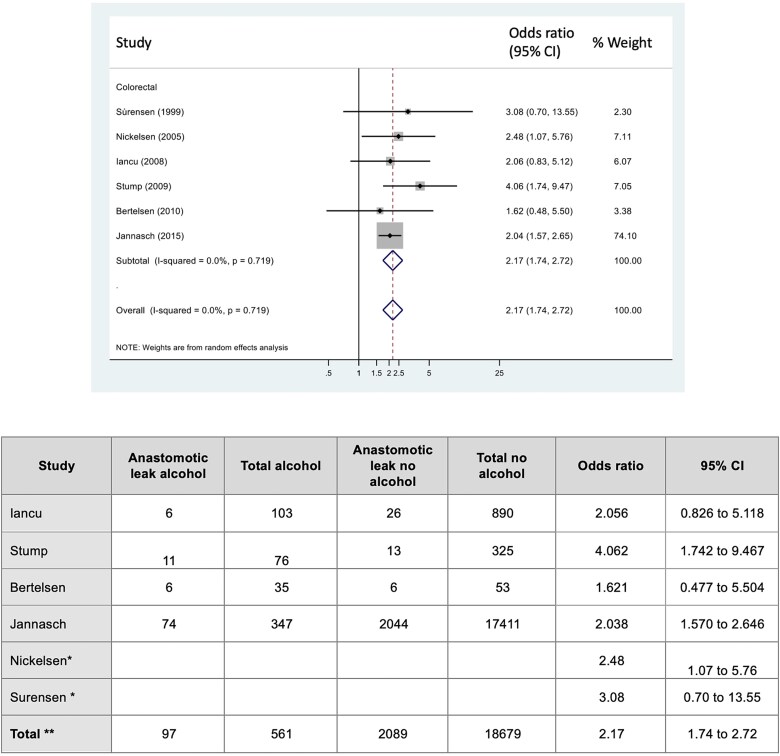
Forest plot for the association between preoperative alcohol consumption and risk of an anastomotic leak. ^*^ denotes studies which report only event rate (Nickelsen et al; and Surensen et al.). ^**^ Total - only include studies that provide actual events; apart from odds ration which is a combined pooled estimate.

Six studies reported on the risk of anastomotic leak (Sørensen et al. 1999; Nickelsen et al. 2005; Iancu et al., 2008; Stumpf et al. 2009; Bertelsen et al. 2010; Jannasch et al. 2015), all in colorectal surgery. The risk of anastomotic leak in the high alcohol intake group was significantly increased following colorectal surgery, compared the no alcohol intake group, OR 2.17 (95% CI: 1.74–2.72, I^2^ = 0%), Fig. 3.

#### Surgical site infections

**Figure 4:**
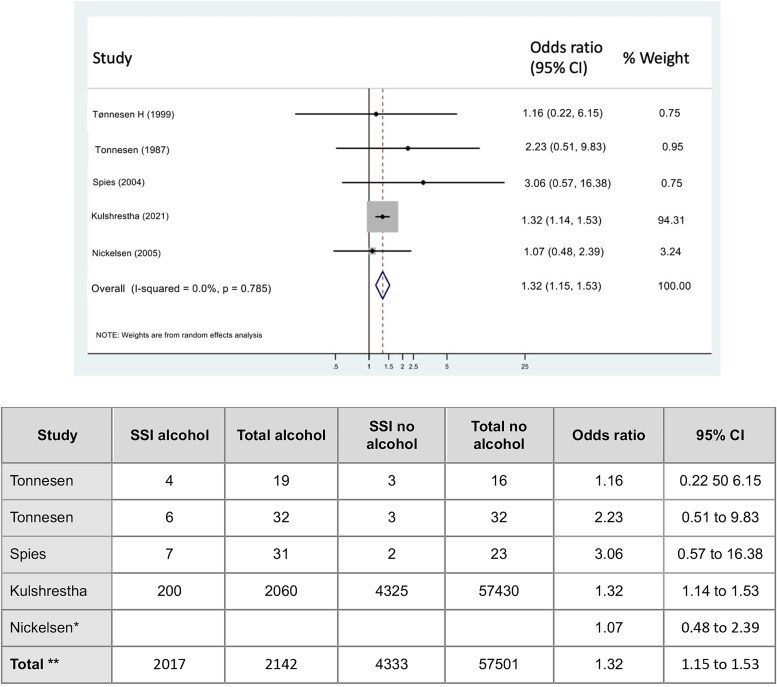
Forest plot for the association between preoperative alcohol consumption and risk of SSI. ^*^ denotes studies which report only event rate (Nickelsen et al; and Surensen et al.). ^**^ Total - only include studies that provide actual events; apart from odds ratio which is a combined pooled estimate.

Finally, 5-studies reported on the risk of surgical site infections (Tønnesen et al. 1987; Tønnesen et al. 1999; Spies et al. 2004; Nickelsen et al. 2005; Kulshrestha et al. 2021). There was an increase in the overall risk of surgical site infections in the high alcohol group, OR = 1.32 (95% CI: 1.15–1.53, I^2^ = 0%), Fig. 4. There were insufficient studies for subgroup analysis.

#### Risk of bias and publication bias

**Figure 5:**
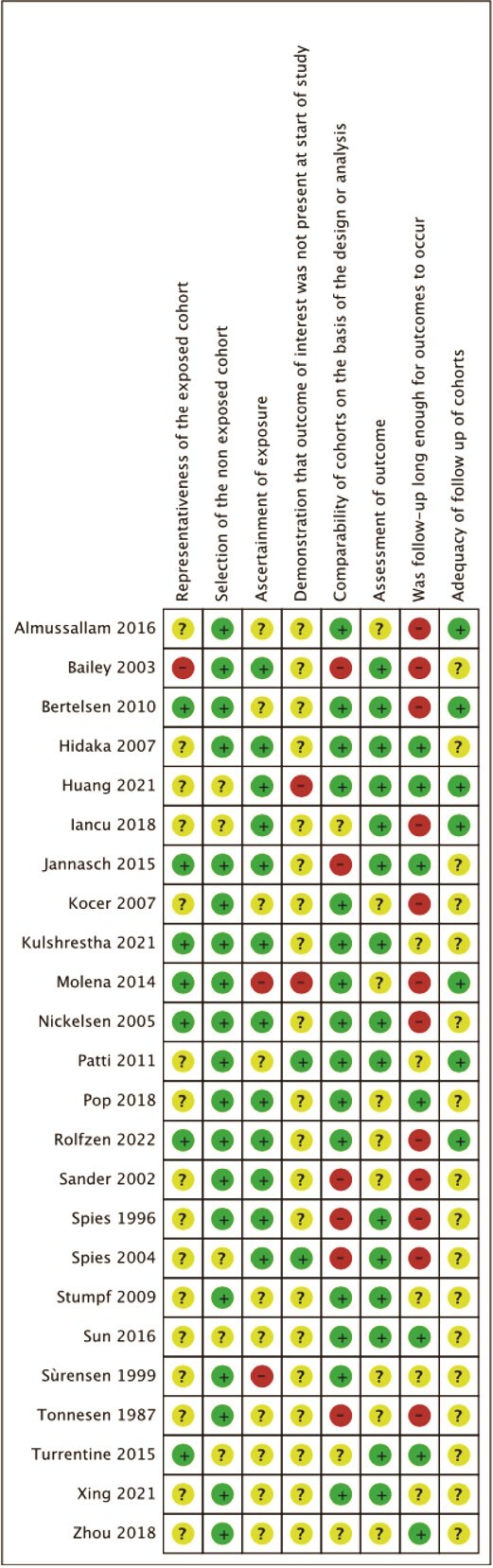
Risk of bias for cohort studies.

**Figure 6:**
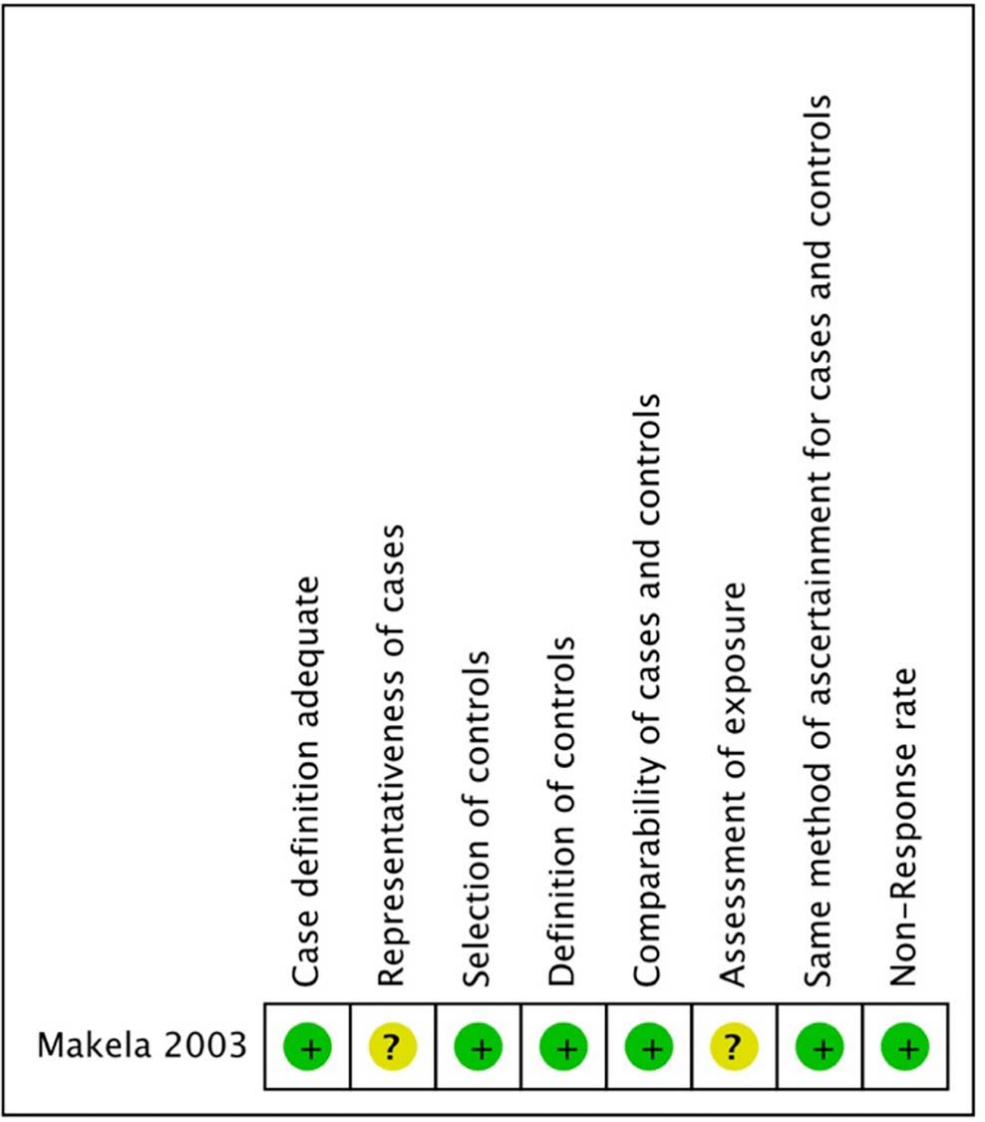
Risk of bias for case control studies.

**Figure 7:**
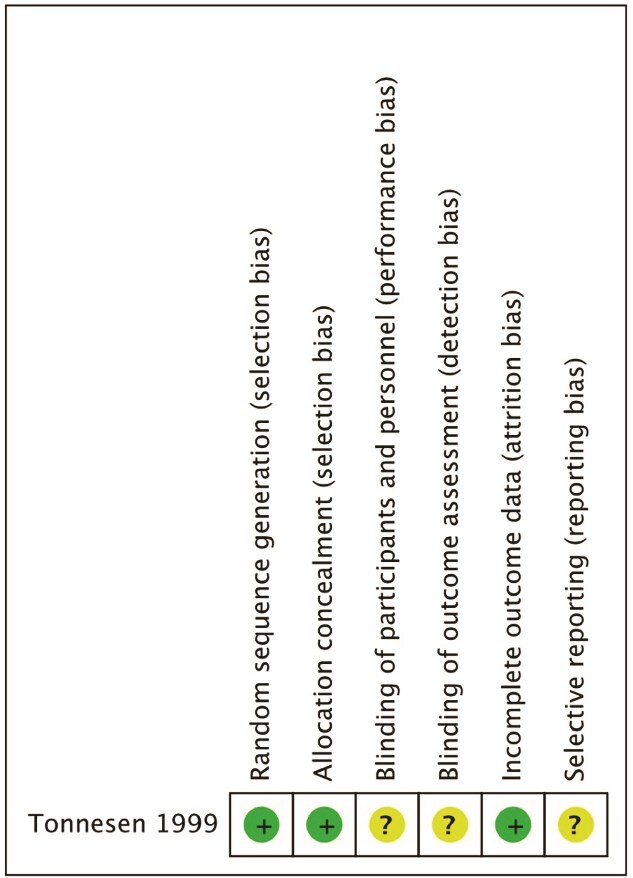
Risk of bias for RCT.

Studies were mostly retrospective and single site in methodology and mostly had a moderate to high risk of bias, Figs 5 and 6. The single RCT was low risk**,**  Fig. 7.

**Figure 8:**
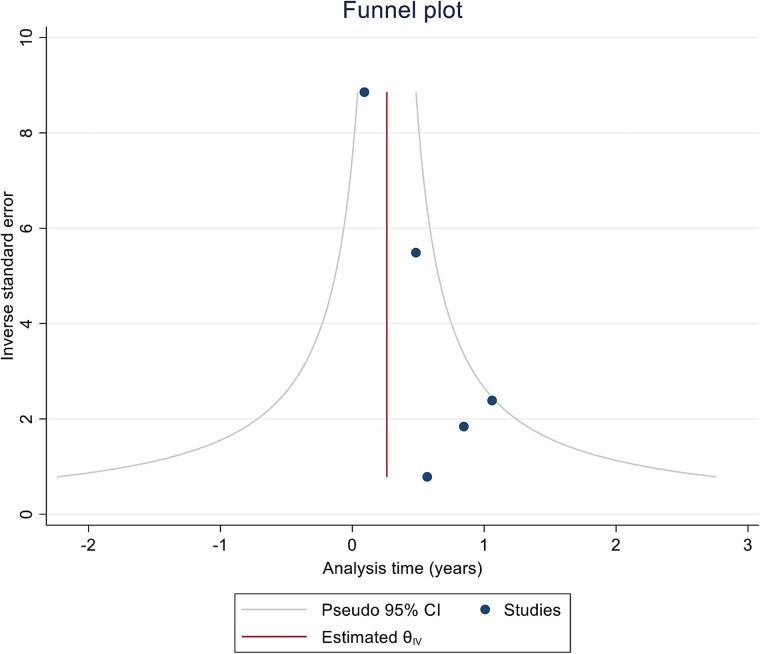
Funnel plot for publication bias.

Publication bias as assessed by visual inspection of the Eggers plot based on the primary outcome showed visual asymmetry – suggesting presence of publication bias (Fig. 8).

## Discussion

### What this study found

There is variability in definitions of high alcohol intake based on study origin and population. Nonetheless, the majority of included studies used a threshold of > 14 units of alcohol intake as a consistent measure of high alcohol use, which allowed for quantitative pooling of results. Patients undergoing gastrointestinal surgery had an overall increased risk of 30-day mortality. On subgroup analysis, it was those patients undergoing colorectal surgery, but not upper GI surgery, who carried the higher mortality burden.

We also identified an overall increased risk of surgical site of infections in patients with high alcohol intake. Our analysis on anastomotic leak was only performed on those patients undergoing colorectal surgery, and we found a similarly increased risk of anastomotic leak in patients with high alcohol intake. This suggests that patients with high alcohol intake have a higher risk of postoperative complications, but they may be particularly vulnerable after colorectal surgery.

### What is already known?

A previous systematic review by Eliasen et al (2013) reporting on 1 234 923 patients undergoing head, abdomen, thorax, orthopaedic and transplant surgical procedures suggested that preoperative alcohol use was associated to an increased risk of general postoperative morbidity, general infections, wound problems, respiratory difficulties, extended hospital stays, and intensive care unit admission (Eliasen et al. 2013). Although that study had considerable clinical and statistical heterogeneity, given the acuity and variability in operative procedures, their findings of higher risk of patients with high alcohol intake align with our findings. (Safe surgery [Internet] 2025).

A population based study using the National Inpatient Sample database, with its reports of in-patient mortality following upper gastrointestinal surgery (total gastrectomy, esophagectomy, total pancreatectomy, or pancreaticoduodenectomy) (Kulshrestha et al. 2021), found that alcohol misuse was linked to higher incidences of postoperative adverse outcomes, longer hospital length of stay (1.54 days [.12, 2.96]) and higher expenses in patients undergoing these major upper gastrointestinal and pancreatic oncologic resections. However, they did not find an association between high alcohol intake and in-patient mortality. The study evaluated mortality during the in-patient stay only, whereas our study examined mortality up to 30-days postoperatively. In our analysis, we found a significant mortality risk following colorectal resections but not after upper gastrointestinal surgery. This could either be due to the low number of studies reporting in upper GI resections or, less likely, that the susceptibility of patients with high alcohol intake differs with different gastrointestinal procedures.

With regards to surgical site infections and anastomotic leak rates, our analysis aligns with the findings of the previous studies showing an increased risk of surgical site infections, high rates of anastomotic leaks following colorectal surgery, and a twofold increased risk of 30-day mortality among individuals who consume high levels of alcohol before surgery when compared to patients who do not drink. Previous studies have also made recommendations for preoperative care pathways that include screening and abstinence programs, and our systematic review supports these recommendations by providing further evidence of the detrimental effects of high preoperative alcohol consumption on postoperative outcomes for patients undergoing gastrointestinal surgery.

### Limitations

The main limitations of this study are related to alcohol consumption definitions and the availability of outcome data. Self-reported alcohol is frequently inaccurate (Livingston and Callinan 2015) due to social desirability bias and recall bias which can lead to underestimations of alcohol consumption. Using more objective measures, such as blood alcohol tests, would have increased the validity of studies and thus our review. Furthermore, there was a lack of consistency in defining high alcohol intake among the included studies, with varying thresholds used. In most cases, this could be standardised by using the the European low risk alcohol intake measure of more than 14 units a week as a threshold, however, this was not possible in all studies.

There was a lack of data on the smoking status of patients in the included studies. Smoking is strongly associated with both alcohol consumption and postoperative complications and is a potential confounding factor in this research (Shiffman and Balabanis 1996) which could not be explored. In addition, we identified moderate statistical heterogeneity in our analysis of 30-day mortality, and accounted for this by undertaking subgroup analysis for this particular outcome. Additionally, 30-day mortality data in US-based studies, except those from the Veterans Affairs system, is often defined as "inpatient" mortality rather than true 30-day mortality, and therefore inpatient mortality is commonly used as a surrogate. This potential limitation should be considered when interpreting our results.

Finally, whilst we identified a growing body of literature evaluating the postoperative outcomes after gastrointestinal surgery in patients with high alcohol intake, the presence of significant clinical and statistical heterogeneity, as well as the inconsistent reporting of outcomes meant that only three outcomes of interest could be evaluated in a meta-analysis. Currently, there are no consensus agreed limits of high alcohol intake leading to variable definitions as identified in our analysis, similarly there are no standardised core outcomes that are required to be reported for studies on alcohol and surgery, and this is an area of research deficiency that would need to be addressed to standardise and improve the quality of outcomes reporting for this group of patients. Despite these limitations and deficiencies in the available data, this study offers unique insights into the risk of complications following gastrointestinal surgery in patients with high alcohol intake.

## Conclusion

Preoperative excessively high alcohol consumption was associated with an increased risk of 30-day mortality, risk of anastomotic leakage and surgical site infections, especially after colorectal surgery. Preoperative modulation of alcohol intake may influence postoperative complications after gastrointestinal surgery.

However, there is a high level of clinical and moderate statistical heterogeneity in the currently available literature necessitating large population-based studies to assess postoperative outcomes after gastrointestinal surgery based on low, moderate or high alcohol intake to further improve our understanding of alcohol impact and role in gastrointestinal surgery. In addition, development of an alcohol specific set of core outcomes following surgery, would allow for better comparisons across different surgical procedures and hospitals, facilitate outcome research, and increase transparency and accountability.

To mitigate the risks associated with preoperative high-risk alcohol use, implementing preoperative screening and intervention programs is essential. These programs can include brief interventions, counselling, and referral to specialised alcohol treatment services to reduce alcohol consumption before surgery. (Fernandez et al. 2015).

## Appendix 1 – Search strategy

1 alcohol.mp. or Alcohols/.

2 (alcohol drinking or Alcohol Drinking).mp. [mp = title, book title, abstract, original title, name of substance word, subject heading word, floating sub-heading word, keyword heading word, organism supplementary concept word, protocol supplementary concept word, rare disease supplementary concept word, unique identifier, synonyms].

3 Alcoholism/ or alcoholic drinking.mp. or Alcohol Drinking/.

4 alcohol abuse.mp. or Alcoholism/.

5 alcohol related disorders.mp. or Alcoholism/ or Alcohol Drinking/ or Alcohol-Related Disorders/.

6 Alcoholics/.

7 alcohol misuse.mp.

8 alcohol intake.mp.

9 harmful drinking.mp.

10 alcohol consumption.mp.

11 alcohol dependent.mp.

12 (alcohol^*^ adj3 (drink^*^ or use^*^ or intake or intervention^*^ or education or program^*^ or misuse^*^ or abuse^*^ or consump^*^)).mp.

13 (drink^*^ adj3 (behaviour or hazardous or harmful^*^ or dependen^*^)).mp. 3609.

14 1 or 2 or 3 or 4 or 5 or 6 or 7 or 8 or 9 or 10 or 11 or 12 or 13.

15 exp Gastrointestinal Stromal Tumors/.

16 exp Upper Gastrointestinal Tract/ or Stomach Neoplasms/.

17 Gastrointestinal Stromal Tumors/ or Gastrointestinal Diseases/ or Gastrointestinal Neoplasms/ or gastrointestinal.mp.

18 exp GASTROINTESTINAL STROMAL TUMORS/ or exp UPPER GASTROINTESTINAL TRACT/ or gastrointestinal.mp. or exp GASTROINTESTINAL NEOPLASMS/ or exp GASTROINTESTINAL TRACT/ or exp GASTROINTESTINAL DISEASES/ or exp LOWER GASTROINTESTINAL TRACT/.

19 Oesophageal.mp.

20 exp Stomach/.

21 exp Gallbladder/.

22 exp Liver/.

23 exp Pancreas/.

24 exp Intestines/.

25 exp Intestinal Obstruction/.

26 exp Intestinal Diseases.

27 exp Intestinal Neoplasms/.

28 small bowel.mp.

29 exp Colon/.

30 exp Rectum/.

31 exp Rectal Neoplasms/.

32 exp Rectal Neoplasms/ or anorectal.mp. or exp Anal Canal/ or exp Rectal Diseases/ or exp ANORECTAL MALFORMATIONS/ or exp Anus Diseases/ or exp Rectum/.

33 surgery.mp. or exp General Surgery/.

34 procedures.mp.

35 exp Surgical Procedures, Operative/.

36 resection.mp.

37 exp surgery/.

38 Operative.mp. or exp SURGICAL PROCEDURES, OPERATIVE/.

39 surgery.mp.

40 operation.mp.

41 15 or 16 or 17 or 18 or 19 or 20 or 21 or 22 or 23 or 24 or 25 or 26 or 27 or 28 or 29 or 30 or 31 or 32.

42 33 or 34 or 35 or 36 or 37 or 38 or 39 or 40.

43 41 and 42.

44 gastrectomy.mp. or exp Gastrectomy/.

45 oesophagectomy.mp.

46 oesophagectomy.mp. or exp Esophagectomy/.

47 Esophagectomy/.

48 exp Bariatric Surgery/ or Gastric Bypass/.

49 exp Cholecystectomy, Laparoscopic/ or exp Cholecystectomy/ or CHOLECYSTECTOMY.mp.

50 Appendectomy/.

51 Appendectomy/ or Appendicitis/ or appendicectomy.mp.

52 hemicolectomy.mp. or exp Colectomy/.

53 anterior resection.mp.

54 abdominoperineal resection.mp.

55 44 or 45 or 46 or 47 or 48 or 49 or 50 or 51 or 52 or 53 or 54.

56 43 or 55.

57 exp Postoperative Complications/.

58 exp perioperative care/.

59 exp preoperative care/.

60 Perioperative Period/.

61 Postoperative Period/.

62 (preoperat^*^ or perioperat^*^ or postoperat^*^ or preoperat^*^ or perioperat^*^ or post operat^*^).mp.

63 complication^*^.mp.

64 exp Patient Readmission/ or readmission.mp. or ‘Length of Stay’/.

65 exp Mortality/.

66 Death/ or death.mp.

67 exp Morbidity/.

68 Clavien-dindo.mp.

69 57 or 58 or 59 or 60 or 61 or 62 or 63 or 64 or 65 or 66 or 67 or 68.

70 case report.tw.

71 letter/.

72 historical article/.

73 Case Study/.

74 case report.tw.

75 abstract report/ or letter/.

76 Conference proceeding.pt.

77 Conference abstract.pt.

78 Editorial.pt.

79 Letter.pt.

80 Note.pt.

81 70 or 71 or 72 or 73 or 74 or 75 or 76 or 77 or 78 or 79 or 80.

82 alcohol^*^.ti,ab.

83 14 and 56 and 69.

84 82 and 83.

85 84 not 81.

## References

[ref1] Almussallam B, Joyce M, Marcello PW. et al. What factors predict hospital readmission after colorectal surgery? - PubMed [internet]. Am Surg. 2016;82:433–8 [cited 2022 Dec 8]. Available from: https://pubmed.ncbi.nlm.nih.gov/27215725/. 27215725

[ref2] Bailey SH, Bull DA, Harpole DH. et al. Outcomes after esophagectomy: a ten-year prospective cohort. Ann Thorac Surg [Internet]. 2003 [cited 2022 Dec 8];75:217–22. Available from: https://pubmed.ncbi.nlm.nih.gov/12537219/. 10.1016/S0003-4975(02)04368-0. 12537219

[ref3] Bertelsen CA, Andreasen AH, Jørgensen T. et al. Anastomotic leakage after anterior resection for rectal cancer: risk factors. Colorectal Dis [Internet]. 2010. [cited 2022 Dec 8];12:37–43. Available from: https://pubmed.ncbi.nlm.nih.gov/19175624/. 10.1111/j.1463-1318.2008.01711.x. 19175624

[ref4] Bode C, Bode JC. Alcohol’s role in gastrointestinal tract disorders. Alcohol Health Res World [Internet]. 1997. [cited 2023 Feb 12];21:76 Available from: /pmc/articles/PMC6826790/. 15706765 PMC6826790

[ref5] Drink less - Better Health - NHS [Internet] . 2025. [cited 2023 Jul 12]. Available from: https://www.nhs.uk/better-health/drink-less/. Published July, 2021.

[ref6] Eliasen M, Grønkjær M, Skov-Ettrup LS. et al. Preoperative alcohol consumption and postoperative complications: a systematic review and meta-analysis. Ann Surg [Internet]. 2013 [cited 2022 Dec 21];258:930–42 Available from: https://journals.lww.com/annalsofsurgery/Fulltext/2013/12000/Preoperative_Alcohol_Consumption_and_Postoperative.13.aspx. 23732268 10.1097/SLA.0b013e3182988d59

[ref7] Fernandez AC, Claborn KR, Borsari B. A systematic review of behavioural interventions to reduce preoperative alcohol use. Drug Alcohol Rev [Internet]. 2015. [cited 2024 Jul 14];34:508. Available from: /pmc/articles/PMC4695381/–20. 10.1111/dar.12285. 26120973 PMC4695381

[ref8] Grossman ER, Benjamin-Neelon SE, Sonnenschein S. Alcohol consumption during the COVID-19 pandemic: a cross-sectional survey of US adults. Int J Environ Res Public Health [Internet]. 2020. [cited 2022 Dec 22];17:1–10. Available from: /pmc/articles/PMC7763183/. 10.3390/ijerph17249189. PMC7763183 33316978

[ref9] Harmful use of alcohol [Internet] . 2025. [cited 2023 Apr 11]. Published Nov 2018. Available from: https://www.who.int/health-topics/alcohol#tab=tab_1.

[ref10] Hidaka H, Hotokezaka M, Nakashima S. et al. Sex difference in survival of patients treated by surgical resection for esophageal cancer. World J Surg [Internet]. 2007. [cited 2022 Dec 8];31:1982–7. Available from: https://pubmed.ncbi.nlm.nih.gov/17676426/. 10.1007/s00268-007-9193-1. 17676426

[ref11] Huang SJ, Zhan PF, Bin CS. Mean corpuscular volume as a prognostic factor for patients with habitual alcohol or tobacco use after esophagectomy. Front Oncol [Internet]. 2021. [cited 2022 Dec 8];11:752229. 10.3389/fonc.2021.752229 Available from: https://pubmed.ncbi.nlm.nih.gov/34868958/. 34868958 PMC8635025

[ref12] Iancu C, Mocan LC, Todea-Iancu D. et al. Host-related predictive factors for anastomotic leakage following large bowel resections for colorectal cancer - PubMed [internet]. J Gastrointestin Liver Dis. 2008, 17:299–303 [cited 2022 Dec 8]. Available from: https://pubmed.ncbi.nlm.nih.gov/18836623/. 18836623

[ref13] Identifying publication bias in meta-analyses of continuous outcomes | Cochrane Training [Internet] . 2025. [cited 2023 Jul 12]. Published July 2020. Available from: https://training.cochrane.org/resource/identifying-publication-bias-meta-analyses-continuous-outcomes#.

[ref14] 9.4.3.0 Introductory text [Internet] . 1999. [cited 2022 Dec 21]. Available from: https://handbook-5-1.cochrane.org/chapter_9/9_4_3_0_introductory_text.htm.

[ref15] Jannasch O, Klinge T, Otto R. et al. Risk factors, short and long term outcome of anastomotic leaks in rectal cancer. Oncotarget [Internet]. 2015. [cited 2022 Dec 8];6:36884. Available from: /pmc/articles/PMC4742217/, 36884–93. 10.18632/oncotarget.5170. 26392333 PMC4742217

[ref16] Kocer B, Surmeli S, Solak C. et al. Factors affecting mortality and morbidity in patients with peptic ulcer perforation. J Gastroenterol Hepatol [Internet]. 2007. [cited 2022 Dec 8];22:565–70 Available from: https://pubmed.ncbi.nlm.nih.gov/17376052/. 17376052 10.1111/j.1440-1746.2006.04500.x

[ref17] Kulshrestha S, Bunn C, Gonzalez R. et al. Unhealthy alcohol and drug use is associated with an increased length of stay and hospital cost in patients undergoing major upper gastrointestinal and pancreatic oncologic resections. Surgery [Internet]. 2021. [cited 2022 Dec 21];169:636–43. Available from: https://pubmed.ncbi.nlm.nih.gov/32951904/. 10.1016/j.surg.2020.07.059. 32951904 PMC7970515

[ref18] Livingston M, Callinan S. Underreporting in alcohol surveys: whose drinking is underestimated? J Stud Alcohol Drugs. 2015;76:158–64. 10.15288/jsad.2015.76.158. 25486405

[ref19] Mäkelä JT, Kiviniemi H, Laitinen S. Risk factors for anastomotic leakage after left-sided colorectal resection with rectal anastomosis. Dis Colon Rectum [Internet]. 2003. [cited 2022 Dec 8];46:653–60 Available from: https://pubmed.ncbi.nlm.nih.gov/12792443/. 12792443 10.1007/s10350-004-6627-9

[ref20] Molena D, Mungo B, Stem M. et al. Incidence and risk factors for respiratory complications in patients undergoing esophagectomy for malignancy: a NSQIP analysis. Semin Thorac Cardiovasc Surg [Internet]. 2014. [cited 2022 Dec 8];26:287–94 Available from: https://pubmed.ncbi.nlm.nih.gov/25837540/. 25837540 10.1053/j.semtcvs.2014.12.002

[ref21] National low-risk drinking recommendations (or drinking guidelines) and standard units | Knowledge for policy [Internet] . 2025. [cited 2023 Jul 12]. Published Nov 2020. Available from: https://knowledge4policy.ec.europa.eu/health-promotion-knowledge-gateway/national-low-risk-drinking-recommendations-drinking-guidelines_en.

[ref22] Nickelsen TN, Jørgensen T, Kronborg O. Lifestyle and 30-day complications to surgery for colorectal cancer. Acta Oncol [Internet]. 2005. [cited 2022 Dec 8];44:218–23. Available from: https://pubmed.ncbi.nlm.nih.gov/16076692/. 10.1080/02841860510029707. 16076692

[ref23] Noncommunicable diseases [Internet] . 2025. [cited 2023 Apr 11]. Published 2021. Available from: https://www.who.int/health-topics/noncommunicable-diseases#tab=tab_1.

[ref24] Patti R, Saitta M, Cusumano G. et al. Risk factors for postoperative delirium after colorectal surgery for carcinoma. Eur J Oncol Nurs [Internet]. 2011. [cited 2022 Dec 8];15:519–23. Available from: https://pubmed.ncbi.nlm.nih.gov/21333597/. 10.1016/j.ejon.2011.01.004. 21333597

[ref25] Pop MG, Fit AM, Vesa SC. et al. Predictors of 1-year postoperative mortality in radical colon cancer surgery - PubMed [internet]. Ann Ital Chir. 2018;89:507–12 [cited 2022 Dec 8]. Available from: https://pubmed.ncbi.nlm.nih.gov/30665223/. 30665223

[ref26] Rolfzen ML, Mikulich-Gilbertson SK, Natvig C. et al. Association between alcohol use disorder and hospital outcomes in colectomy patients - a retrospective cohort study. J Clin Anesth [Internet]. 2022. [cited 2022 Dec 8];78: Available from: https://pubmed.ncbi.nlm.nih.gov/35168136/. 10.1016/j.jclinane.2022.110674.35168136

[ref27] Safe surgery [Internet] . 2025. [cited 2023 Jan 9]. Published 2021. Available from: https://www.who.int/teams/integrated-health-services/patient-safety/research/safe-surgery.

[ref28] Sander M, Irwin M, Sinha P. et al. Suppression of interleukin-6 to interleukin-10 ratio in chronic alcoholics: association with postoperative infections. Intensive Care Med [Internet]. 2002. [cited 2022 Dec 8];28:285–92 Available from: https://pubmed.ncbi.nlm.nih.gov/11904657/. 11904657 10.1007/s00134-001-1199-9

[ref29] Shiffman S, Balabanis M. Do drinking and smoking go together? Alcohol Health Res World [Internet]. 1996. [cited 2022 Dec 26];20:107 Available from: /pmc/articles/PMC6876501/. 31798093 PMC6876501

[ref30] Sørensen LT, Jørgensen T, Kirkeby LT. et al. Smoking and alcohol abuse are major risk factors for anastomotic leakage in colorectal surgery. Br J Surg [Internet]. 1999. [cited 2022 Dec 8];86:927–31. Available from: https://pubmed.ncbi.nlm.nih.gov/10417567/. 10.1046/j.1365-2168.1999.01165.x. 10417567

[ref31] Spies CD, Nordmann A, Brummer G. et al. Intensive care unit stay is prolonged in chronic alcoholic men following tumor resection of the upper digestive tract. Acta Anaesthesiol Scand [Internet]. 1996. [cited 2022 Dec 8];40:649–56. Available from: https://pubmed.ncbi.nlm.nih.gov/8836256/. 10.1111/j.1399-6576.1996.tb04505.x. 8836256

[ref32] Spies CD, Von Dossow V, Eggers V. et al. Altered cell-mediated immunity and increased postoperative infection rate in long-term alcoholic patients. Anesthesiology [Internet]. 2004. [cited 2022 Dec 8];100:1088–100. Available from: https://pubmed.ncbi.nlm.nih.gov/15114205/. 10.1097/00000542-200405000-00010. 15114205

[ref33] Stumpf M, Junge K, Wendlandt M. et al. Risk factors for anastomotic leakage after colorectal surgery. Zentralbl Chir [Internet]. 2009. [cited 2022 Dec 8];134:242–8 Available from: https://pubmed.ncbi.nlm.nih.gov/19536719/. 19536719 10.1055/s-0028-1098773

[ref34] Sun P, Chen C, Zhang F. et al. Combined heavy smoking and drinking predicts overall but not disease-free survival after curative resection of locoregional esophageal squamous cell carcinoma. Onco Targets Ther [Internet]. 2016. [cited 2022 Dec 8];Volume 9:4257–64. Available from: https://pubmed.ncbi.nlm.nih.gov/27471400/. 10.2147/OTT.S104182. 27471400 PMC4948733

[ref35] Tønnesen H, Schütten BT, Jørgensen BB. Influence of alcohol on morbidity after colonic surgery. Dis Colon Rectum [Internet]. 1987. [cited 2022 Dec 8];30:549–51 Available from: https://pubmed.ncbi.nlm.nih.gov/3595376/. 3595376 10.1007/BF02554788

[ref36] Tønnesen H, Rosenberg J, Nielsen HJ. et al. Effect of preoperative abstinence on poor postoperative outcome in alcohol misusers: randomised controlled trial. BMJ [Internet]. 1999. [cited 2022 Dec 8];318:1311–6. Available from: https://pubmed.ncbi.nlm.nih.gov/10323814/. 10.1136/bmj.318.7194.1311. 10323814 PMC27867

[ref37] Tsuchiya H . Anesthetic effects changeable in habitual drinkers: mechanistic drug interactions with neuro-active indoleamine-aldehyde condensation products associated with alcoholic beverage consumption. Med Hypotheses [Internet]. 2016. [cited 2022 Dec 22];92:62–6. Available from: https://pubmed.ncbi.nlm.nih.gov/27241259/. 10.1016/j.mehy.2016.04.038. 27241259

[ref38] Turrentine FE, Denlinger CE, Simpson VB. et al. Morbidity, mortality, cost, and survival estimates of gastrointestinal anastomotic leaks. J Am Coll Surg [Internet]. 2015 Feb. [cited 2022 Dec 8];220:195–206 Available from: https://pubmed.ncbi.nlm.nih.gov/25592468/. 25592468 10.1016/j.jamcollsurg.2014.11.002

[ref39] Xing J, Liu M, Qi X. et al. Risk factors for esophagojejunal anastomotic leakage after curative total gastrectomy combined with D2 lymph node dissection for gastric cancer. J Int Med Res [Internet]. 2021. [cited 2022 Dec 8];49. Available from: https://pubmed.ncbi.nlm.nih.gov/33736508/. 10.1177/03000605211000883. PMC7983250 33736508

[ref40] Zhou C, Wu XR, Liu XH. et al. Male gender is associated with an increased risk of anastomotic leak in rectal cancer patients after total mesorectal excision. Gastroenterol Rep (Oxf) [Internet]. 2018. [cited 2022 Dec 8];6:137. Available from: /pmc/articles/PMC5952946/–43. 10.1093/gastro/gox039. 29780603 PMC5952946

